# Validating a clinical prediction score for *Legionella*-related community acquired pneumonia

**DOI:** 10.1186/s12879-022-07433-z

**Published:** 2022-05-09

**Authors:** Rosalie R. A. L. Beekman, Ruud R. Duijkers, Dominic D. Snijders, Menno M. van der Eerden, Martijn M. Kross, Wim W. G. Boersma

**Affiliations:** 1 Department of Pulmonology, Noordwest Ziekenhuisgroep, Alkmaar, Netherlands; 2 Department of Pulmonology, Medisch Centrum Leeuwarden, Henri Dunantweg 2, 8934 AD Leeuwarden, Netherlands; 3 Department of Pulmonology, Spaarne Ziekenhuis, Hoofddorp, Netherlands; 4 Department of Pulmonology, Erasmus MC, Rotterdam, Netherlands; 5 Department of Pulmonology, Slotervaartziekenhuis, Amsterdam, Netherlands

**Keywords:** *Legionella*, Community-acquired pneumonia, Clinical prediction rule, Rapid diagnosis

## Abstract

**Background:**

*Legionella-*related community acquired pneumonia (CAP) is a disease with an increasing incidence and a high mortality rate, especially if empirical antibiotic therapy is inadequate. Antibiotic treatment highly relies on clinical symptoms, although proven non-specific, because currently available diagnostic techniques provide insufficient accuracy for detecting Legionella CAP on admission. This study validates a diagnostic scoring system for detection of *Legionella*-related CAP, based on six items on admission (Legionella prediction score).

**Methods:**

We included patients with *Legionella*-related CAP admitted to five large Dutch hospitals between 2006 and 2016. Controls were non-*Legionella*-related CAP patients. The following six conditions were rewarded one point if present: fever > 39.4 °C; dry cough; hyponatremia (sodium) < 133 mmol/L; lactate dehydrogenase (LDH) > 225 mmol/L; C-reactive protein (CRP) > 187 mg/L and platelet count < 171 × 10^9^/L. The accuracy of the prediction score was assessed by calculating the area under the curve (AUC) through logistic regression analysis.

**Results:**

We included 131 cases and 160 controls. A score of 0 occurred in non-*Legionella-*related CAP patients only, a score of 5 and 6 in *Legionella-*related CAP patients only. A cut-off ≥ 4 resulted in a sensitivity of 58.8% and a specificity of 93.1%. The AUC was 0.89 (95% CI 0.86–0.93). The strongest predictors were elevated LDH, elevated CRP and hyponatremia.

**Conclusions:**

This multi-centre study validates the Legionella prediction score, an easily applicable diagnostic scoring system, in a large group of patients and finds high diagnostic accuracy. The score shows promise for future prospective validation and could contribute to targeted antibiotic treatment of suspected *Legionella* CAP.

## Introduction

*Legionella* infection is an important cause of community-acquired pneumonia (CAP) with a mortality of 8–12% [1]. The average incidence of *Legionella* infection in CAP was reported 2.1–3.6% in a recent meta-analysis [2]. However, due to underdiagnosis, the true incidence is probably higher. In the USA, the incidence of reported *Legionella* cases quadrupled over the past decades [3–6]. *Legionella*-related pneumonia has an overall higher burden of morbidity and mortality than other causes of CAP, especially if initial empirical antibiotic therapy is inadequate [3, 5, 7, 8].

Diagnosis of *Legionella-*related CAP is difficult, because culturing *Legionella* from sputum and blood takes 3 to 10 days and has a low yield. The introduction of the urinary antigen test (UAT) for *Legionella pneumophila* improved diagnosis, especially in severe cases. However, the UAT can be negative in the early phase of the disease, especially in patients with mild disease. UAT detects only *Legionella pneumophila* serogroup 1 antigens, accounting for more than 80% of *Legionella* cases [9–11]. Over the last years several other subspecies of *Legionella* have been associated with significant clinical disease, especially *Legionella longbeachae* which is predominantly found in Australia, New Zealand and Asia, but recently has been detected in both USA and Europe [12–14].

To prevent overuse of macrolides and quinolones, international guidelines recommend empirical antibiotic coverage of *Legionella* only when this infection is suspected based on clinical signs and symptoms, or in patients with severe CAP. Clinical scoring systems were developed to predict *Legionella*-related pneumonia, but most have limited clinical significance because of low accuracy or the need to include follow-up data over several days [15–17]. As a result initial empirical coverage may be inadequate [18–20].

Fiumefreddo et al. [21] developed a diagnostic scoring system consisting of 6 items which are easily obtainable on admission, namely fever, dry cough, hyponatremia, elevated lactate dehydrogenase (LDH) and elevated C-reactive protein (CRP), further called: Legionella prediction score. In the derivation cohort, the diagnostic accuracy of the score was high, with an area under the curve (AUC) of 0.86 (95% confidence interval (CI) 0.81–0.90) [21]. In a previous validation study with 37 cases, the Legionella prediction score discriminated reasonably well between *Legionella*-related CAP and CAP caused by other pathogens (specificity 92% and sensitivity 31% at cut off ≥ 4, area under the curve 0.91) [22].

In theory this prediction score could be a useful clinical tool to limit antibiotic overuse in selected patients, especially in cases that are not detected by UAT, due to a false-negative result, when UAT takes too long or it has not been performed. Therefore, we evaluated the performance of this score through external validation in a large, Dutch cohort of hospital-admitted patients with *Legionella*-related CAP.

## Methods

### Patients and materials

In this cross-sectional, observational, retrospective study, data was collected from four large teaching hospitals and one University hospital in the Netherlands. A list of all patients tested positive with *Legionella* species between 2006 and 2016 was provided by the departments of microbiology. Medical records of all patients were reviewed and data was collected anonymously. Cases had at least one microbiological test positive for *Legionella* species, either culture, serology, PCR or UAT and consolidation(s) of the chest X-ray. UATs were performed with the BinaxNOW *S. pneumoniae* Antigen Card (Abbott). Our control group consisted of non-*Legionella* CAP-patients who required hospital admission, through random selection of participants from the REDUCE study, which was conducted in one of the previously mentioned teaching hospitals from 2013–2017 (full study protocol available via clinicaltrials.gov, NCT01964495). All patients included in the study had at least one consolidation on the chest X-ray together with clinical signs and symptoms indicative for CAP. Other inclusion criteria were a (pre-event) life expectancy of minimum 30 days and granted informed consent. Patients were excluded if they were pregnant or breastfeeding, if they had immunodeficiency (known immunodeficiency or receiving corticosteroids equivalent to 10 mg prednisolone per day), cancer, in case of obstruction, aspiration or hospital acquired pneumonia and if they were unable or unlikely to comprehend and follow the REDUCE protocol. Patients were included in the analysis if the items needed to calculate the Legionella prediction score were present. When available, causative pathogens for non-*Legionella* CAP were reported. Causative pathogens were defined as pathogens known to cause CAP, that were isolated from blood cultures, sputum cultures of good quality sputum (predominant leukocytes without squamous epithelial cells), urinary antigen tests and/or PCR. We retrospectively collected data from patient files which were anonymized for our study before processing the data. Therefore, in accordance to the current guidelines at that time in the Netherlands, informed consent was not required, neither was approval by a local ethics committee. This conforms to the Law regarding Medical Research involving human subjects in the Netherlands.

### Data and Legionella prediction score

The collected data included vital parameters, clinical signs, laboratory findings, relevant comorbidities, smoking history and CURB-65 score. All data was obtained during the emergency care department visit (further called: on admission). The Legionella prediction score, ranging from 0–6, was calculated. For the six following conditions, if present, one point was scored: fever > 39.4 °C; dry cough; hyponatremia (sodium) < 133 mmol/L; LDH > 225 mmol/L; high CRP > 187 mg/L and low platelet count < 171 × 10^9^/L [21].

### Outcomes

The primary outcome was the diagnostic accuracy of the Legionella prediction score for *Legionella*-related CAP. Furthermore, we assessed the predictive value of the original, continuous parameters and the proposed cut-off points, both univariate and multivariate.

### Statistical analysis

According to Toll et al., for validation of a prediction rule with a dichotomous outcomes, at least 100 cases and 100 controls are needed [23]. We estimated that a random selection of 185 REDUCE participants would provide sufficient controls. Patient characteristics were assessed for normal distribution with the Kolmogorov–Smirnov test. Either mean, standard deviation and Chi-square test or median, percentiles and Mann–Whitney-U test were reported.

Continuous parameters were analysed in a logistic regression model that was performed for each individual parameter and for all parameters combined. Thereafter, parameters were dichotomized in the categories used in the prediction score. Univariate and multivariate logistic regression was repeated with these dichotomized parameters.

For each regression, the *b-*coefficient, the odds ratio, AUC and *p* were calculated. A *p*-value < 0.05 was considered significant. Sensitivity, specificity, positive and negative predictive values were calculated for scores 0–6 of the prediction score.

To further assess accuracy, chi-square, the loglikelihood ratio and Nagelkerke square were calculated. IBM SPSS Statistics version 25.0 was used for all analyses.

## Results

**Fig. 1:**
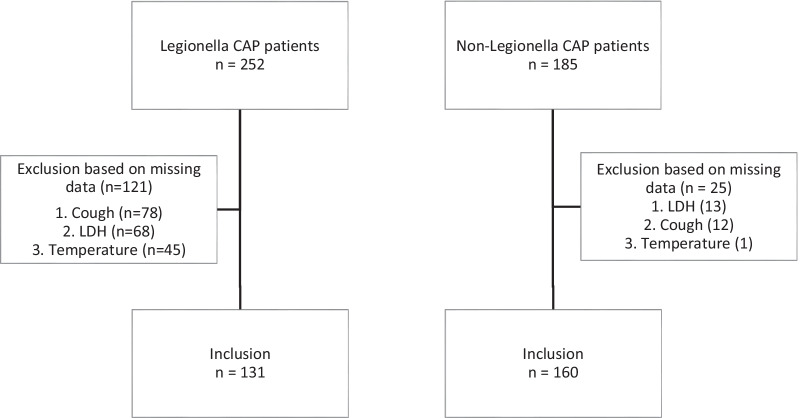
Flow chart of inclusion and exclusion. Patients were excluded if items needed to calculate the Legionella prediction score could not be obtained. These items are: temperature, dry cough, sodium, LDH, CRP and platelets. *CAP* community acquired pneumonia, *LDH* lactate dehydrogenase, *CRP* C-reactive protein

We identified 252 patients with *Legionella-*related CAP and 185 patients with non-*Legionella* related CAP. Of 252 patients with *Legionella*, 131 had complete data and were included. Of non-*Legionella* patients, 160 were included as controls (Fig. 1). Baseline characteristics are summarized in Table 1. Patients with *Legionella* were often male, relatively younger, had less comorbidities (such as COPD and cancer), but were more frequently active smokers. Of cases, 126 (96%) were confirmed by UAT and 29 (22%) were confirmed by sputum PCR or culture. In the control group, the most frequently detected pathogens were *Streptococcus pneumoniae* (18.8%), *Staphylococcus aureus* (10.1%) and *Haemophilus influenzae* (8.1%). A further specification of pathogens in the control group is available in Table 2.

**Table 1:** Patient characteristics

	*Legionella* CAP n = 131	Non-*Legionella* CAP n = 160	p
Male, n (%)	88 (67.2)	89 (55.6)	0.037
Age [years], median (IQR)	63.4 (56.2–70.3)	72.2 (64.7–81.4)	< 0.001
Current smoker, n (%)	43 (53.8)	29 (20.1)	< 0.001
Comorbidities, n (%)			
COPD	11 (8.4)	47 (29.4)	< 0.001
Congestive heart failure	11 (8.4)	21 (13.1)	0.22
Neurologic disease	15 (11.5)	37 (23.1)	0.012
Cancer	8 (6.1)	33 (20.6)	< 0.001
Renal disease	4 (3.1)	15 (9.4)	0.033
Liver disease	4 (3.1)	2 (1.3)	0.271
Symptoms, n (%)			
Dyspnea	79 (68.7)	124 (83.8)	0.005
Dry cough	77 (58.8)	50 (31.3)	< 0.001
Headache	31 (66.0)	14 (32.6)	< 0.001
Muscle or joint pain	37 (64.9)	25 (51.0)	0.143
Nausea	36 (52.9)	28 (48.3)	0.538
Vomiting	28 (41.8)	20 (37.0)	0.686
Diarrhea	42 (53.2)	13 (15.5)	< 0.001
Confusion	24 (18.3)	32 (20.0)	0.718
Physical findings, median (IQR)			
Heart frequency [BPM]	100 (82–112)	96 (83–111)	0.635
Systolic blood pressure (mmHg)	130 (118–145)	135.5 (119–151)	0.139
Diastolic blood pressure (mmHg)	73 (65–81)	75.5 (67–83)	0.111
Body temperature (°C)	39 (37.8–39.7)	38.4 (37.5–39.1)	0.002
CURB-65 [score 0–5], median (IQR)	1 (0–2)	2 (1–2)	0.016
Previous antibiotic treatment, n (%)	39 (33.6)	42 (26.8)	0.226
Laboratory findings, median (IQR)			
Urea (mmol/L)	7.1 (5.5–11.4)	7.2 (5.0–9.1)	0.333
Creatinine (mc mol/L)	101 (80–131)	88 (66–108)	< 0.001
Sodium (mmol/L)	132 (129–135)	136 (133–138)	< 0.001
Potassium (mmol/L)	3.9 (3.5–4.2)	3.9 (3.6–4.3)	0.186
Bilirubin (µmol/L)	12 (8–19)	13 (8–16)	0.628
ASAT (IU/L)	49 (33–103)	26 (19–35)	< 0.001
ALAT (IU/L)	36 (24–67)	19 (13–29)	< 0.001
LDH (IU/L)	465 (324–609)	198 (168–238)	< 0.001
AP (IU/L)	79 (69–115)	82 (67–113)	0.242
GGT (IU/L)	47 (30–79)	37 (24–67)	0.034
C-reactive protein (mg/L)	317 (244–390)	162 (77–260)	< 0.001
Hemoglobin (mmol/L)	8.3 (7.8–9.1)	8.1 (7.3–8.9)	0.067
Platelets (× 10^9^/L)	204 (156–249)	221 (179–293)	0.002
White blood cell count (× 10^9^/L)	13 (10–17)	13 (10–18)	0.377

*CAP* community acquired pneumonia, *IQR* interquartile range, *BPM* beats per minute, *ASAT* aspartate transaminase, *ALAT* alanine transaminase, *LDH* lactate dehydrogenase, *AP* alkaline phosphatase, *GGT* gamma-glutamyltransferase

**Table 2:** Pathogens detected in participants with non-Legionella community acquired pneumonia

Pathogen	n	(%)
*S. pneumoniae*	30	(18.8)
*S. aureus*	16	(10.0)
*H. influenzae*	13	(8.1)
Influenza A Virus	11	(6.9)
*E. coli*	9	(5.6)
*M. pneumoniae*	7	(4.4)
*P. aeruginosa*	7	(4.4)
*M. catarrhalis*	5	(3.1)
Rhinovirus	4	(2.5)
*K. pneumoniae*	4	(2.5)
Coronavirus	3	(1.9)
*H. parainfluenzae*	2	(1.3)
Other	6	(3.8)
None	74	(44.6)

In univariate regression of the original values (Table 3), all six predictors were significantly associated with *Legionella-*related CAP. The strongest predictors were sodium, CRP and LDH levels (AUC respectively 0.76, 0.80 and 0.93). In multivariate regression, this association persisted for all parameters except for dry cough. The AUC of the multivariate model of these variables was 0.96 (95% CI 0.94–0.98).

**Table 3:** Univariate and multivariate analysis of the different predictors

	Univariate analysis				Multivariate analysis		
	B	OR (95% CI)	p	AUC (95% CI)	B	OR (95% CI)	p
Temperature	0.29	1.33 (1.08–1.64)	0.007	0.61 (0.54–0.67)	0.508	1.66 (1.15–1.64)	0.007
Dry cough	1.14	3.14 (1.94–5.08)	< 0.001	0.64 (0.57–0.70)	0.640	1.90 (0.83–5.08)	0.128
Sodium	− 0.215	0.81 (0.76–0.86)	< 0.001	0.76 (0.70–0.81)	− 0.144	0.87 (0.79–0.86)	0.002
LDH	0.016	1.02 (1.01–1.02)	< 0.001	0.93 (0.90–0.96)	0.015	1.02 (1.01–1.02)	< 0.001
CRP	0.009	1.01 (1.01–1.01)	< 0.001	0.80 (0.75–0.85)	0.008	1.01 (1.00–1.01)	< 0.001
Platelets	− 0.005	1.00 (0.99–1.00)	0.002	0.61 (0.54–0.67)	− 0.005	1.00 (0.99–1.00)	0.046

*OR* odds ratio, *CI* confidence interval, *AUC* area under the curve, *LDH* lactate dehydrogenase, *CRP* C-reactive protein

In Table 4 all variables were expressed as dichotomous parameters. In univariate analysis again, all predictive values were statistically significant. The strongest predictors were hyponatremia < 133 mmol/L, elevated CRP > 187 mg/L and elevated LDH > 225 mmol/L (AUC respectively 0.71, 0.75 and 0.81). In the multivariate model, dry cough was a significant predictor. Fever above 39.4 °C and platelets below 171 × 10^9^/L were not significant predictors. The AUC of the complete dichotomic multivariate model was 0.89 (95% CI 0.86–0.93).

**Table 4:** Univariate and multivariate analysis of the dichotomized variables

	Univariate analysis				Multivariate analysis		
	B	OR (95% CI)	p	AUC (95% CI)	B	OR (95% CI)	p
Temperature > 39.4 °C	0.77	2.17 (1.24–3.78)	0.006	0.57 (0.50–0.63)	0.45	1.56 (0.66–3.70)	0.311
Dry cough		*See Table **3*			0.82	2.28 (1.10–4.73)	0.027
Sodium < 133 mmol/L	1.84	6.27 (3.72–10.58)	< 0.001	0.71 (0.64–0.77)	1.44	4.24 (1.98–9.08)	< 0.001
LDH > 225 mmol/L	3.74	42.1 (17.4–101.7)	< 0.001	0.81 (0.76–0.86)	3.42	30.54 (11.3–82.4)	< 0.001
CRP > 187 mg/L	2.64	14.0 (7.3–26.9)	< 0.001	0.75 (0.70–0.81)	2.23	9.29 (4.11–21.03)	< 0.001
Platelets < 171 × 10^9^/L	0.56	1.76 (1.03–3.01)	0.039	0.55 (0.49–0.62)	0.70	2.02 (0.88–4.65)	0.099

*OR* odds ratio, *CI* confidence interval, *AUC* area under the curve, *LDH* lactate dehydrogenase, *CRP* C-reactive protein

**Fig. 2:**
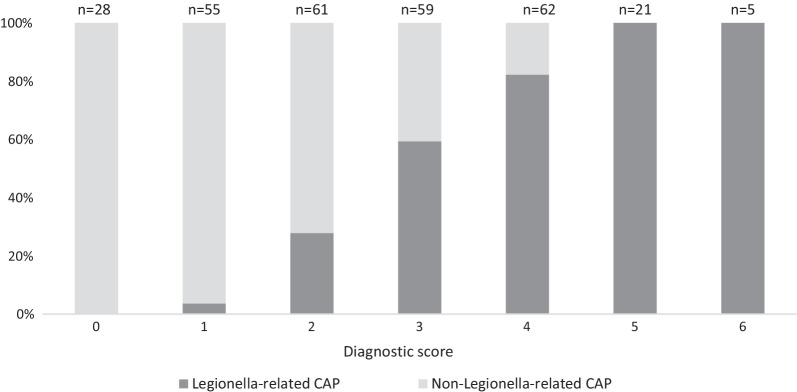
Distribution of participants per score. This figure shows the percentage of Legionella cases (dark grey) and controls (light grey) per possible outcome of the Legionella prediction score (0 to 6 points). N: total number of participants with this score

**Fig. 3:**
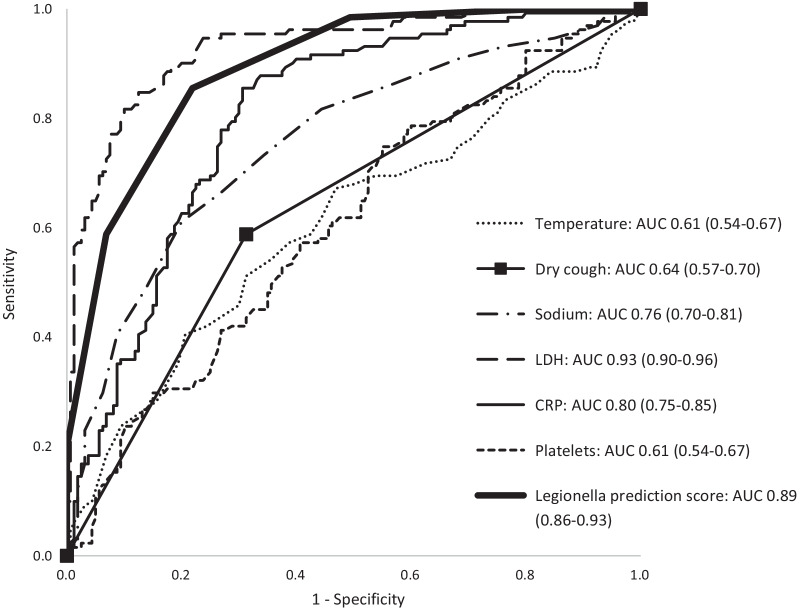
ROC-curve of individual parameters and Legionella predictive score. This figure shows the ROC-curve of the individual parameters, analyzed as continuous values. Furthermore, it shows the ROC-curve of the diagnostic scoring system, which is calculated by dichotomizing the individual parameters, followed by multivariate regression analysis. *ROC-curve* receiver operating curve, *AUC* area under the curve, *LDH* lactate dehydrogenase, *CRP* C-reactive protein

As shown in Fig. 2, a prediction score of 0 only occurred in non-*Legionella-*related CAP patients. Above, the number of cases gradually increased per score point. A prediction score of 5 or 6 points was only found in *Legionella-*related CAP patients (specificity 100%). The prediction score detected *Legionella* with a specificity of 93.1% and a sensitivity of 58.8% when a cut-off ≥ 4 was chosen. A cut-off ≥ 2 resulted in a sensitivity of 98.5% and a specificity of 50.6%. Figure 3 illustrates the receiver operating characteristics curve (ROC-curve) of the individual predictors and of the prediction score.

## Discussion

*Legionella-*related CAP is a disease with a high mortality rate and increasing incidence [1–6]. It requires targeted antibiotic treatment, in an era where antibiotic resistance is rising and antibiotic stewardship is important. Although clinical symptoms of *Legionella* prove non-specific [15]*,* they can be a decisive factor in the treatment choice on admission [18–20]*.* This retrospective study further validated a prediction score based on six clinical parameters, that can be applied easily on admission, and found a high accuracy with an AUC of 0.89 (95% CI 0.86–0.93). We demonstrated that this score can potentially be used to rule-in or rule-out Legionella CAP, depending on the cut-off point chosen. Therefore, in patients presenting with mild to moderate disease symptoms, it could be applied both for early identification and specific treatment of those infected with *Legionella*, in particular in cases that are not detected by UAT. The negative predictive value of the score will likely be higher in an unselected population of hospital admitted CAP patients, since the incidence of Legionella is lower than in our population.

All predictors were associated significantly with the outcome. However, temperature and platelets were no significant predictors in the multivariate analysis after dichotomization. Assumably, this can be explained by the wide range in which these variables occurred in both patients with *Legionella* CAP and with non-*Legionella* CAP.

Our study yielded an accuracy similar to that found by a Spanish study (AUC 0.86 (95% CI 0.81–0.90)), based on 82 cases [21]. It was higher than in a previous multinational validation study, which found an AUC of 0.73 (95% CI 0.65–0.81) [22]. This difference can be explained by a smaller sample size (37 cases). Baseline differences between the cases and controls (age, COPD and smoking) in our study were similar to both other studies [22]. This was not the case in a Japanese validation study published in 2017, in which participants were more often male and that also included patients with cancer [24]. They found a sensitivity of 94% and a specificity of 49% at a cut-off ≥ 2, resembling our present study.

In the literature two other diagnostic scoring systems for *Legionella*-related CAP were proposed, namely the Winthrop University score and the Community-Based Pneumonia Incidence Study Group scoring system. These two scoring systems were validated, but found unsuitable for diagnosing or excluding Legionella in a clinical setting, due to low accuracy [16, 17, 24, 25].

A Japanese study group recently proposed a variation on the Legionella prediction score, which includes dyspnoea and gender instead of on temperature and platelets. This score performed well (AUC 0.93) in a Japanese validation cohort. However, in study populations outside Japan, male gender and dyspnoea were not identified as risk factors for *Legionella*-related CAP. Therefore this score may be less relevant [26].

This multi-centre study included a large number of patients with *Legionella-*related CAP. The number of participants considerably exceeds the number that is due sufficient for validation of a prediction score with a dichotomous outcome, according to Toll et al. [23]. All hospital admitted patients with CAP were eligible for inclusion and data was collected from five different large hospitals with a wide geographical spread. This adds to the external validity of the study because it closely resembles a real-life clinical population. We chose to only include patients with complete data, so imputation of missing data could be avoided which adds on to the internal validity of the study. However, this has the potential to introduce some sort of selection bias but given the large sample of patients we believe the effect of this potential bias is likely small.

A weak point of this study is that its retrospective. Missing data on occurrence of especially (dry) cough lead to many exclusions. In a prospective study setting, this parameter would be easy to obtain. Furthermore, cases were retrospectively selected, based on positive microbiological tests. Mostly, this was the UAT, which does not detect species other than *Legionella pneumophila* serogroup 1. Because cultures and PCR have not been performed in all participants, some *Legionella* cases might have been missed. This could potentially influence the performance of the score. A Japanese study demonstrated a better performance of the Legionella prediction score for *Legionella* serogroup 1 (N = 11) than for other *Legionella* species (n = 23) [27]. This suggests that the score is particularly useful for detecting *Legionella* serogroup 1, which was detected in 96% of the cases in the present study.

Future research should validate the diagnostic scoring system prospectively, preferably in an unselected CAP population, in which *Legionella* is detected via UAT, PCR and cultures. This research could also analyse the accuracy of the scoring system, give more insight into performance of the score over the course of the disease, mild versus advanced disease, and investigate its clinical significance in addition to UAT. Moreover, longitudinal studies on clinical outcomes resulting from implementation of the test, such as change in antibiotic prescriptions, mortality, ICU admissions and of length of stay in the hospital, are needed.

## Conclusion

This six-items prediction score detects Legionella related CAP infections with a high specificity of 93.1% (sensitivity 58.8%) in patients who score positive for at least four items. It is easy to implement in day to day practice with data readily available in every CAP patient and. Overall, based on our data and previous studies we believe it shows promise for further prospective validation and could contribute to targeted antibiotic treatment of *Legionella-*related CAP.

## Data Availability

The datasets used and/or analysed during the current study are available from the corresponding author on reasonable request.

## Abbreviations

ALAT: Alanine transaminase
AP: Alkaline phosphatase
ASAT: Aspartate transaminase
AUC: Area under the curve
BPM: Beats per minute
CAP: Community-acquired pneumonia
CI: Confidence interval
COPD: Chronic obstructive pulmonary disease
CRP: C-reactive protein
GGT: Gamma-glutamyltransferase
IQR: Interquartile range
LDH: Lactate dehydrogenase
OR: Odds ratio
PCR: Polymerase chain reaction
ROC-curve: The receiver operating characteristics curve
UAT: Urinary antigen test
